# Performance of large-volume plasma PCR for the diagnosis of invasive aspergillosis: a multicenter retrospective cohort study

**DOI:** 10.1128/jcm.00703-26

**Published:** 2026-09-25

**Authors:** Hanne Lamberink, Ga-Lai M. Chong, Sammy Huygens, Mickey Konings, Thomas de Corte, Wendy W. J. van de Sande, Corné H. W. Klaassen, Bart J. A. Rijnders

**Affiliations:** 1 Department of Internal Medicine, Section of Infectious Diseases, Erasmus MC University Medical Center 6993, Rotterdam, the Netherlands; 2 Department of Medical Microbiology and Infectious Diseases, Erasmus MC University Medical Center 6993, Rotterdam, the Netherlands; 3 Department of Hematology, Sint-Jan Brugge-Oostende General Hospital 60208 , Bruges, Belgium; 4 Department of Hematology, Ghent University Hospital 60200 , Ghent, Belgium; 5 Department of Internal Medicine and Pediatrics, Faculty of Medicine and Health Sciences, Ghent University 26656, Ghent, Belgium; University of Calgary, Calgary, Alberta, Canada

**Keywords:** invasive pulmonary aspergillosis, invasive fungal infection, polymerase chain reaction, plasma

## Abstract

Timely diagnosis of invasive aspergillosis (IA) is hindered by moderate sensitivity of conventional diagnostic methods. Increasing plasma input volumes has been shown to improve sensitivity of plasma polymerase chain reaction (PCR), and using plasma-derived fungal exosomes (nanosized extracellular vesicles) may do the same. This study aimed to determine whether using large plasma input volumes and exosome-derived DNA enhances the diagnostic performance of *Aspergillus* PCR. We included hematology patients from whom plasma was collected within 48 h of bronchoalveolar lavage in the prospective multicenter ARPO-study (NCT06069505); plasma from healthy donors served as controls. Sensitivity, specificity, positive predictive values, and negative predictive values of the PCR on DNA extracted from plasma-derived exosomes, large plasma volumes of around 4.0 mL, and small plasma volumes of 0.2 mL were assessed using the European Organization for Research and Treatment of Cancer/Mycoses Study Group Education and Research Consortium diagnostic criteria. The IA prevalence was 34.2% (38/111), and the median plasma volume used in the large-volume group was 4.0 mL (IQR 3.1 mL–4.0 mL). *Aspergillus fumigatus* large-volume plasma PCR yielded 22 positive results with a sensitivity of 52.6%, significantly higher than with small plasma volume (29.7%, *P* = 0.039) without loss of specificity (97.2%), improving further in patients not receiving versus receiving mold-active antifungals (66.7% versus 40.0%, *P* = 0.013). Sensitivity of the PCR on plasma-derived exosomes was low (29.4%). *A. flavus* DNA was detected in five patients and three healthy controls; all were considered false positives. DNA extraction from larger plasma volumes improved the detection of *A. fumigatus*. The sensitivity of plasma-derived exosomes was limited; their use is not recommended.

**IMPORTANCE:**

Invasive fungal infection caused by *Aspergillus fumigatus* remains a life-threatening complication in patients with hematological malignancies, while early diagnosis is often challenging with the current tools. This study demonstrates that increasing the volume of blood plasma used for fungal DNA detection significantly improves diagnostic sensitivity without compromising specificity. As blood sampling is less invasive than bronchoalveolar lavage, a more sensitive plasma-based approach may facilitate earlier detection and initiation of targeted therapy when utilizing a diagnostic-driven approach. Our findings provide practical guidance for optimizing molecular diagnostic protocols in clinical laboratories.

## INTRODUCTION

Invasive aspergillosis (IA) is a major cause of morbidity and mortality in patients with hematological malignancies, highlighting the critical need for accurate, timely diagnosis. Conventional diagnostic methods, including fungal culture and galactomannan (GM) detection, are limited by suboptimal sensitivity and specificity, potentially delaying the initiation of appropriate antifungal therapy.

Molecular diagnostics, particularly polymerase chain reaction (PCR) assays targeting *Aspergillus* DNA, have emerged as promising alternatives. The updated European Organization for Research and Treatment of Cancer/Mycoses Study Group Education and Research Consortium (EORTC/MSGERC) criteria now include two consecutive positive PCR tests on bronchoalveolar lavage (BAL) fluid, serum, plasma, and/or whole blood as a mycological criterion for IA (1), reflecting growing confidence in PCR testing and the ongoing debate regarding the clinical significance of a single positive result (2, 3).

PCR is more commonly performed on BAL fluid than blood, with sensitivities of 77%–88% and specificities of 75%–95% (4, 5). However, blood-based PCR testing could offer an attractive, less invasive alternative and may provide a substantially earlier diagnostic signal (6). Previous meta-analyses report sensitivities of 79%–88% and specificities of 75%–80% for a single blood PCR, whereas ≥2 PCR tests yielded sensitivities of 60%–75% and specificities of 87%–95% (7, 8). Plasma PCR in hematological patients showed higher sensitivity than serum PCR (91%–95% vs 68%–80% [6, 9]) and serum GM testing (86% vs 68% [10]), and is less affected by mold-active prophylaxis, although its specificity may decrease (7, 10–13). Despite these advantages, lack of standardization across PCR assays limits comparability due to differences in extraction protocols, target genes, and amplification platforms. The European *Aspergillus* PCR Initiative established optimized protocols for testing whole blood (14), serum (15), and plasma (16), showing that larger input volumes, among other factors, improve PCR sensitivity (10, 17–21), with an optimized volume of 4 mL (13).

We further sought to explore exosomes as a novel target for minimally invasive PCR diagnostics. Exosomes are nanosized extracellular vesicles secreted by host cells and microbes, including *Aspergillus fumigatus* and *Aspergillus flavus* (22–25), which mediate intercellular communication and host-microbe interactions via transfer of proteins, lipid particles, and DNA (26, 27). Building on advances in virology (28, 29) and oncology (30, 31), we conducted a pilot study to evaluate whether utilizing plasma-derived exosomes, along with optimizing plasma PCR input volume, could improve the sensitivity of a validated in-house *Aspergillus* PCR assay in patients with hematological malignancies and suspected invasive aspergillosis.

## MATERIALS AND METHODS

### Study design and patient population

The objective was to evaluate the diagnostic yield of an in-house *Aspergillus* PCR, using larger plasma volumes and plasma-derived exosomes, compared to small plasma volumes. Plasma samples were obtained from the prospective multicenter Azole Resistance PCR Optimization Study (NCT06069505), which includes high-risk patients with hematological malignancies undergoing BAL for a suspected invasive fungal disease, with concordant abnormalities on high-resolution chest CT (HRCT). Serum and plasma were typically collected in a 48-h window around the time of BAL. The three participating study sites were tertiary care centers and followed a diagnostic-driven strategy for antifungal therapy, rather than an empirical approach. Mold-active antifungal prophylaxis was administered during remission-induction therapy for acute myeloid leukemia (AML) at one center, and to patients with severe acute or chronic graft-versus-host disease at all centers.

To ensure accurate classification according to EORTC/MSGERC 2020 criteria, we excluded patients in whom important routine diagnostic tests on BAL were missing or incomplete (e.g., GM testing and/or PCR not performed, or only a single positive PCR result available). Consequently, all patients classified as possible IA had a negative GM and PCR on BAL and, when performed, also on serum, thereby reducing misclassification of indeterminate cases and ensuring a fully evaluable non-IA comparator group for diagnostic accuracy assessment. Additionally, patients who had received antifungal therapy for >5 days at the time of plasma sampling were excluded. We considered proven and probable IA as true cases, and possible and no IA controls. Healthy plasma donors from the Convalescent Plasma as Therapy for Severe SARS-CoV-2 Disease Study (CONCOVID, NCT04342182) served as additional negative controls.

Our study received ethical approval from the institutional review boards at each site, and written informed consent was obtained from all patients.

### Data collection

Clinical data were obtained from electronic patient records, including details on hematological disease, immunosuppressive conditions, HRCT abnormalities, antifungal prophylaxis and treatment, and 6-week survival. Microbiological data from routine diagnostics were retrieved from laboratory information management systems.

### Microbiological testing and classification of IA

BAL was performed according to local standards, with routine mycological testing including direct microscopy, fungal culture, ELISA GM antigen testing (Platelia, Bio-Rad, Hercules, CA, USA), and *Aspergillus* PCR with either the AsperGenius 1.0 PCR or an in-house PCR. Patients were classified based on EORTC/MSGERC 2020 criteria as proven, probable, possible, or no IA. We considered *A. fumigatus* the causative agent when *A. fumigatus* had been identified on BAL by culture or by PCR. In patients with a positive BAL GM but negative BAL PCR, we also considered *A. fumigatus* the most likely pathogen due to its 80%–90% prevalence reported in Europe (32, 33). Similarly, when *A. flavus* or *A. terreus* was cultured, or in the case of the latter identified with a dedicated *A. terreus* PCR probe, we considered *A. flavus* or *A. terreus* the causative pathogen.

### AsperGenius 1.0 and the in-house *Aspergillus* PCR

The AsperGenius 1.0 multiplex PCR (PathoNostics, Maastricht, the Netherlands) detects the most prevalent *Aspergillus* species by targeting the multicopy 28S rRNA gene and has dedicated probes for *A. fumigatus* and *A. terreus* (but not *A. flavus*). This commercially available assay was used at the participating centers on BAL samples as part of routine clinical care according to the manufacturer’s instructions (version 3, 2015 [34]).

In addition, a validated in-house multiplex PCR targeting the ITS2 region was performed, detecting the most prevalent *Aspergillus* species (*A. fumigatus*, *A. flavus,* and *A. terreus*). PCR reactions (20 µL) contained 5 µL isolated DNA, primers, and probes (see Table 1 for sequences and concentrations) in 1× PerfeCTa multiplex mix (VWR, Amsterdam, the Netherlands). An initial activation/denaturation step at 95°C was followed by 50 cycles of denaturation at 95°C for 5 seconds and annealing/extension at 60°C for 30 seconds, after which the reactions were cooled down. To monitor the entire process, an internal control with phocine herpesvirus (PhHV) was used. DNA extraction from small plasma volumes of 0.2 mL was done with the MagNA Pure 96 System (Roche Diagnostics, Almere, the Netherlands); the elution volume was 50 µL. DNA extraction from 4.0 mL (or at least 1.0 mL when less than 4.0 mL was available) was done with the Maxwell RSC Instrument with the Maxwell RSC ccfDNA Plasma Kit (Promega Corporation, Madison, WI, USA); the elution volume was 75 µL. To assess the efficiency of DNA extraction from large plasma volumes, human β-globin was measured. As opposed to lower cut-off values typically used for BAL fluid, cycle threshold values (Ct-values) ≤ 40.0 were considered positive as no plasma colonization or contamination is expected.

**TABLE 1:** The sequences of the primers and probes used in the in-house *Aspergillus* PCR to target the most prevalent *Aspergillus* species

	Fwd primer (5′−3′)	Probe (label-sequence-quencher)	Rev primer (5′−3′)
*A. fumigatus*	GTATGGGGCTTTGTCACCTG (0.5 µM)	FAM-CGCCAGCCGACACCCAACTT-EDQ (0.1 µM)	CCTACCTGATCCGAGGTCAA (0.5 µM)
*A. flavus*	GGCTTTGTCACCCGCTCTGT (0.5 µM)	Cy5-GCTTGCCGAACGCAAATCA-BHQ2 (0.1 µM)	
*A. terreus*	GTATGGGGCTTCGTCTTCC (0.5 µM)	TexasRed-CGCCGACGCATTTATTTGCAAC-BHQ2 (0.1 µM)	
PhHV	GGGCGAATCACAGATTGAATC (0.5 µM)	YakimaYellow-TTTTTATGTGTCCGCCACCATCTGGATC-EDQ (0.1 µM)	GCGGTTCCAAACGTACCAA (0.5 µM)

### Exosome enrichment

Exosomes were isolated from 1.0 mL plasma with the miRCURY Exosome Isolation Kit – Serum and Plasma (Qiagen, Hilden, Germany) according to the manufacturer’s protocol (35). Thrombin (10 µL) was added to 1.0 mL of plasma and incubated for 5 minutes at room temperature (15°C–25°C), followed by centrifugation at 10,000 × *g* for 5 minutes. The supernatant (800 µL) was mixed with Precipitation Buffer A (320 µL) and incubated for 60 minutes at 4°C, followed by centrifugation at 500 × *g* for 5 minutes at 20°C. The pellet was resuspended in 250 µL, resulting in a final volume of around 300 µL. PCR was then performed on DNA extracted from the exosomes using the MagNA Pure 96 System.

### Statistical analysis

Non-parametric continuous variables were summarized as medians (IQR) and compared using the Mann-Whitney *U*-test. Categorical variables were compared using χ² or Fisher’s exact tests, as appropriate. Diagnostic performance was assessed by sensitivity, specificity, and positive and negative predictive values (PPV and NPV, respectively), with paired comparisons by McNemar’s test. The optimal Ct-value cut-off for 6-week mortality was determined from the receiver operating characteristic (ROC) curve using the Kolmogorov-Smirnov (KS) metric and Youden’s *J* statistic. DNA extraction and amplification efficiency were assessed by comparing predicted and actual β-globin Ct-values after adjustment for elution volumes. *P* < 0.05 was considered statistically significant. Analyses were performed using IBM SPSS Statistics version 28.0.1.0 and GraphPad Prism version 10.6.0.

## RESULTS

### Study population

**Fig 1:**
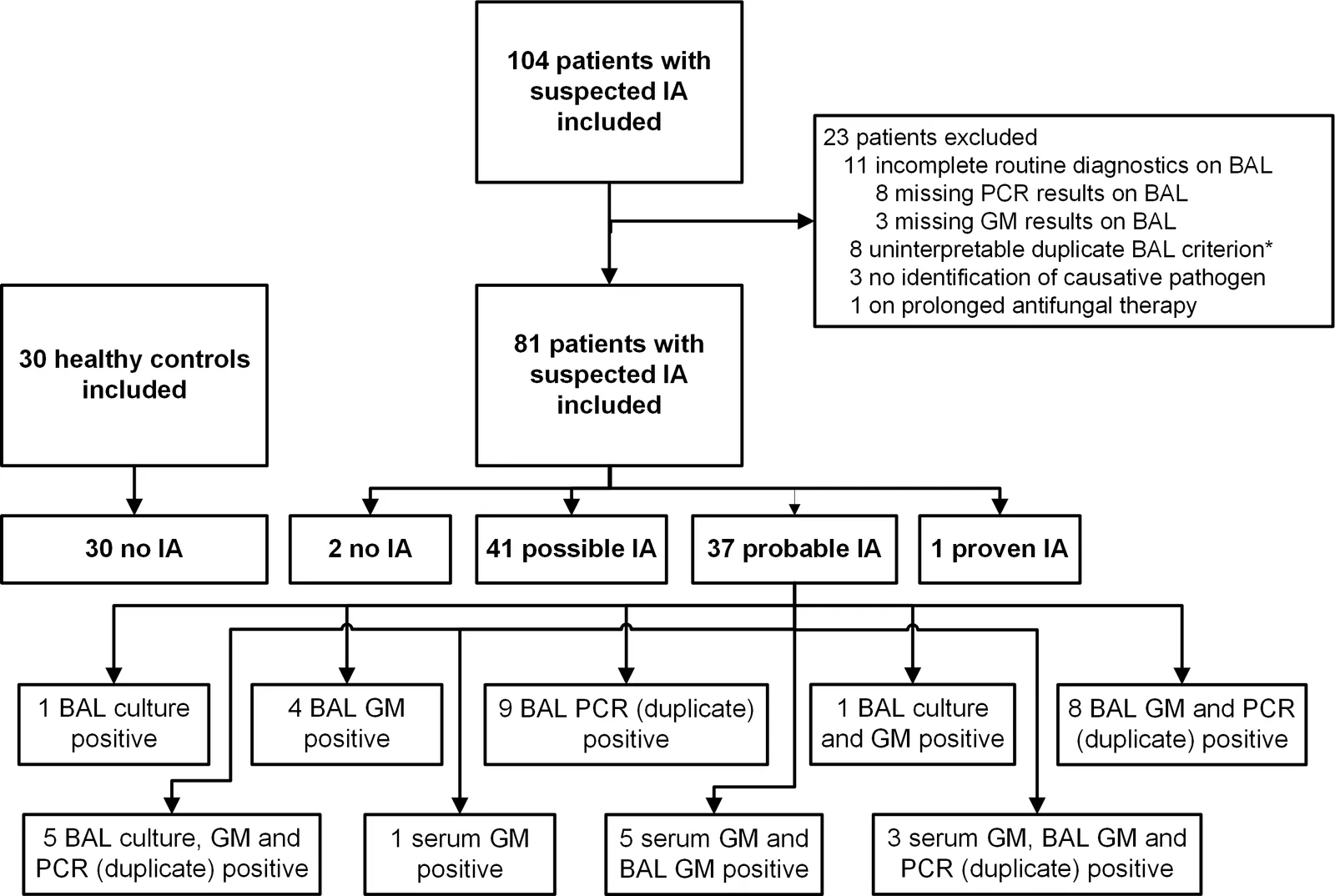
Flowchart of patients, including reasons for exclusion and, for patients classified as having probable invasive aspergillosis, the mycological criteria met during routine diagnostic workup.

We included 134 samples from 104 hospitalized patients with underlying hematological disease and suspected IA, and 30 healthy controls. For the purpose of this study, 23 hematology patients were excluded from the analysis: 11 because of incomplete routine diagnostics (3 missing BAL GM, 8 missing BAL PCR), 8 because duplicate PCR results were unavailable, 3 because the causative pathogen could not be determined, and 1 because of prolonged antifungal therapy (Fig. 1). The duplicate PCR criterion was considered uninterpretable when resistance PCR was not performed (e.g., when only *Aspergillus* species but not *Aspergillus fumigatus* was detected) or when amplification failed for both targets, TR34/L98H and TR46/T289A/Y121F. Baseline characteristics are shown in Table 2. Of the 81 patients at risk for IA, 1 had proven IA (1.2%), 37 probable IA (45.7%), 41 possible IA (50.6%), and 2 had no IA (2.5%). The prevalence of IA (proven and probable IA) in the at-risk hematology patients was 46.9% (38/81) and 34.2% (38/111) in all subjects. In all 38 cases, the causative pathogen was classified as *A. fumigatus* (8 by culture, 20 with the *A. fumigatus* PCR, 10 by galactomannan alone); *A. flavus* or *A. terreus* were not detected. All healthy controls were considered not to have IA.

**TABLE 2:** Baseline characteristics of 81 at-risk patients included^*a*^^,^^*g*^

	All patients (*N* = 81) Median (IQR) or *n* (%)
Age, years	64.0 (56.5–70.0)
Sex, male	54 (66.7)
Underlying hematological malignancy	
AML	37 (45.7)
MDS	11 (13.6)
ALL	4 (4.9)
Other	29 (35.8)
Neutropenia^*b*^	51 (63.0)
Use of immunosuppressants	32 (39.5)
High-dose corticosteroids	8 (9.9)
T-cell immunosuppressants	7 (8.6)
B-cell immunosuppressants	6 (7.4)
Combination	11 (13.6)
Allogeneic HSCT	20 (24.7)
CAR-T therapy	1 (1.2)
Antifungal therapy or prophylaxis^*c*^	49/79 (62.0)
Fluconazole	32/79 (40.5)
Mold-active AFT	17/79 (21.5)
Voriconazole	6/79 (7.6)
Posaconazole	1/79 (1.3)
L-AmB	1/79 (1.3)
Combination	9/79 (11.4)
Positive fungal diagnostics on BAL	
Microscopy^*d*^	3 (3.7)
Fungal culture	13 (16.0)
*A. fumigatus*	8 (9.9)
*Rhizopus microsporus*	1 (1.2)
*Candida* species	4 (4.9)
ELISA GM ODI	0.3 (0.1–2.0)
ELISA GM (≥1.0)^*e*^	25 (30.9)
*Aspergillus* PCR (in duplicate)	26 (32.1)
Positive fungal diagnostics on serum^*f*^	
ELISA GM ODI	0.1 (0.0–0.2)
ELISA GM (≥0.5)	13 (16.0)
EORTC/MSGERC 2020 classification	
Proven IA	1 (1.2)
Probable IA	37 (45.7)
Possible IA	41 (50.6)
No IA	2 (2.5)

^*a*^ If information was missing, we reported the denominator of patients for whom the data were known. The baseline characteristics of the 30 negative controls were unknown as no clinical data were collected for these subjects. All 30 negative controls were considered not to have IA.

^*b*^ ≤0.5 × 10^9^/L at time of plasma sampling (±48 h).

^*c*^ In the last 48 h before plasma sampling.

^*d*^ Microscopic detection of fungal elements in BAL fluid indicating a mold.

^*e*^ BAL GM tested with the Platelia assay.

^*f*^ Serum ELISA GM positivity with the Platelia assay based on results from either the local laboratory or from the central lab used in the VirCLIA GM test validation study (PMC11837514) when available.

^*g*^ AFT, antifungal therapy; ALL, acute lymphoblastic leukemia; AML, acute myeloid leukemia; BAL, bronchoalveolar lavage; CAR-T, chimeric antigen receptor T-cell therapy; ELISA, enzyme-linked immunosorbent assay-based galactomannan testing (Platelia); EORTC/MSGERC, European Organization for Research and Treatment of Cancer and the Mycoses Study Group Education and Research Consortium; GM, galactomannan; HSCT, hematopoietic stem cell transplantation; IA, invasive aspergillosis; L-AmB, liposomal amphotericin B; MDS, myelodysplastic syndrome; ODI, optical density index; PCR, polymerase chain reaction.

### Diagnostic accuracy of PCR on large plasma volume

Large-volume plasma PCR was performed on plasma from 81 patients and 30 healthy controls (see Fig. S1 for the samples tested); the median plasma volume used for DNA extraction was 4.0 mL (IQR 3.1–4.0). The test result of one sample was excluded due to a negative internal control. For the large-volume *A. fumigatus* PCR*,* there were 22/110 (20.0%) positive results with a sensitivity of 52.6% (20/38), specificity of 97.2% (70/72), PPV of 90.9% (20/22), and NPV of 79.5% (70/88). A *post hoc* sensitivity analysis comparing diagnostic accuracy parameters for probable/proven IA versus possible/no IA, and probable/proven IA versus no IA, was performed; see Table 3. For *A. flavus*, 8 of 110 (7.3%) samples tested positive with a median Ct-value of 35.3 (IQR 33.1–36.0): 5 of 80 (6.3%) patients and 3 of 30 (10%) controls. All 8 were considered false positives, as *A. flavus* was not detected in BAL fluid of any of the 5 patients with probable or proven invasive aspergillosis. Without true cases and only false-positive results, no further analysis was conducted. For *A. terreus*, there were no positive test results.

**TABLE 3:** Overview of diagnostic accuracy parameters for the in-house *A. fumigatus* PCR and the impact of disease prevalence, different definitions of the absence of IA, and the exclusion of data of the healthy controls in the analysis^*a*^

	Prevalence of IA	Sensitivity (%)	Specificity (%)	PPV (%)	NPV (%)	LR +	LR –
Proven/probable IA versus possible/no IA, all subjects (patients at risk of IA and healthy controls)							
Large plasma volume	38/110 (34.5%)	52.6	97.2	90.9	79.5	18.9	0.49
Proven/probable IA versus no IA, all subjects (patients at risk of IA and healthy controls)							
Large plasma volume	38/70 (54.3%)	52.6	100.0	100.0	64.0	Not estimable	0.47
Proven/probable IA versus possible/no IA, only patients at-risk of IA							
Large plasma volume	38/80 (47.5%)	52.6	95.2	90.9	69.0	11.1	0.50
Small plasma volume	37/80 (46.3%)	29.7	95.3	84.6	61.2	6.4	0.74
Plasma-derived exosomes	17/25 (68.0%)	29.4	100.0	100.0	40.0	Not estimable	0.71
Proven/probable IA versus no IA, only patients at-risk of IA							
Large plasma volume	38/40 (95.0%)	52.6	100.0	100.0	10.0	Not estimable	0.47
Small plasma volume	37/39 (94.9%)	29.7	100.0	100.0	7.1	Not estimable	0.70
Plasma-derived exosomes	17/19 (89.5%)	29.4	100.0	100.0	14.3	Not estimable	0.71
Modeled PPV and NPV of large plasma volume with assumed prevalences of IA, all subjects							
High-risk setting	(20%)	52.6	97.2	82.6	89.1	18.9	0.49
Screening setting	(5%)	52.6	97.2	49.9	97.5		
Modeled PPV and NPV of large plasma volume with assumed prevalences of IA, only patients at risk for IA							
High-risk setting	(20%)	52.6	95.2	73.4	88.9	11.05	0.50
Screening setting	(5%)	52.6	95.2	36.8	97.4		

^*a*^ The first sensitivity analysis shows the impact of the use of the EORTC possible/no IA criteria for defining the absence of IA rather than the EORTC no IA criteria only; the second sensitivity analysis shows the impact of excluding the healthy controls, leaving only the at-risk hematology patients. *A. fumigatus* PCR was performed on large plasma volume (Mdn 4.0 mL) with the Maxwell RSC Instrument, on small plasma volume (0.2 mL) with the MagNA Pure 96 System, and on plasma-derived exosomes with the MagNA Pure 96 System (from 1.0 mL plasma).

### Influence of antifungal use on performance of large-volume plasma PCR

Data on antifungal use at least 72 h before plasma sampling were available for 93/110 (84.5%) subjects, assuming the healthy controls did not receive antifungal therapy. When stratified by the use of at least 72 h of mold-active antifungals at the time of plasma sampling, the sensitivity of large-volume plasma *A. fumigatus* PCR was significantly higher (McNemar’s *P* = 0.013) in those without antifungals (sensitivity 66.7%, specificity 98.4%, PPV 93.3% and NPV 89.6% for *n* = 82) compared to those with antifungals (sensitivity 40.0%, specificity 100%, PPV 100% and NPV 66.7% for *n* = 11).

### Diagnostic performance of PCR on small plasma volume

PCR results on small plasma volume (0.2 mL) were available for the 81 patients with suspected IA. The test result of one sample was excluded due to a negative internal control. The *A. fumigatus* PCR was positive in 13/80 samples (16.3%), with a sensitivity of 29.7% (11/37), a specificity of 95.3% (41/43), a PPV of 84.6% (11/13), and an NPV of 61.2% (41/67). For *A. flavus*, 3/80 samples tested positive (3.8%), all considered false positive; further analysis was therefore futile. For *A. terreus*, 1/80 samples tested positive (1.3%) and was also considered false positive.

### Comparison of *A. fumigatus* PCR performance on large versus small plasma volume

Including all samples on which the *A. fumigatus* PCR was performed with available results for both large and small volume, the sensitivity for large plasma volume (52.6%, 20 true positives of 38 IA cases) was significantly higher (McNemar’s *P* = 0.039) than the sensitivity for small plasma volume (29.7%, 11 true positives of 37 IA cases; one internal control was not successful).

### Efficiency of large-volume plasma PCR

β-Globin DNA Ct-values were available from 32 samples from at-risk patients for both large and small plasma volume PCR. The median predicted decrease in Ct-values using large plasma volumes instead of small plasma volumes was 3.3 (IQR 3.1–3.7), also considering different elution volumes. The median observed decrease was 4.3 (IQR 3.3–4.7), *P* < 0.001, meaning that the increase in volume was indeed at least inversely proportional to the Ct-values of the internal control; see Table S1.

### Ct-value cut-off and clinical outcome

**Fig 2:**
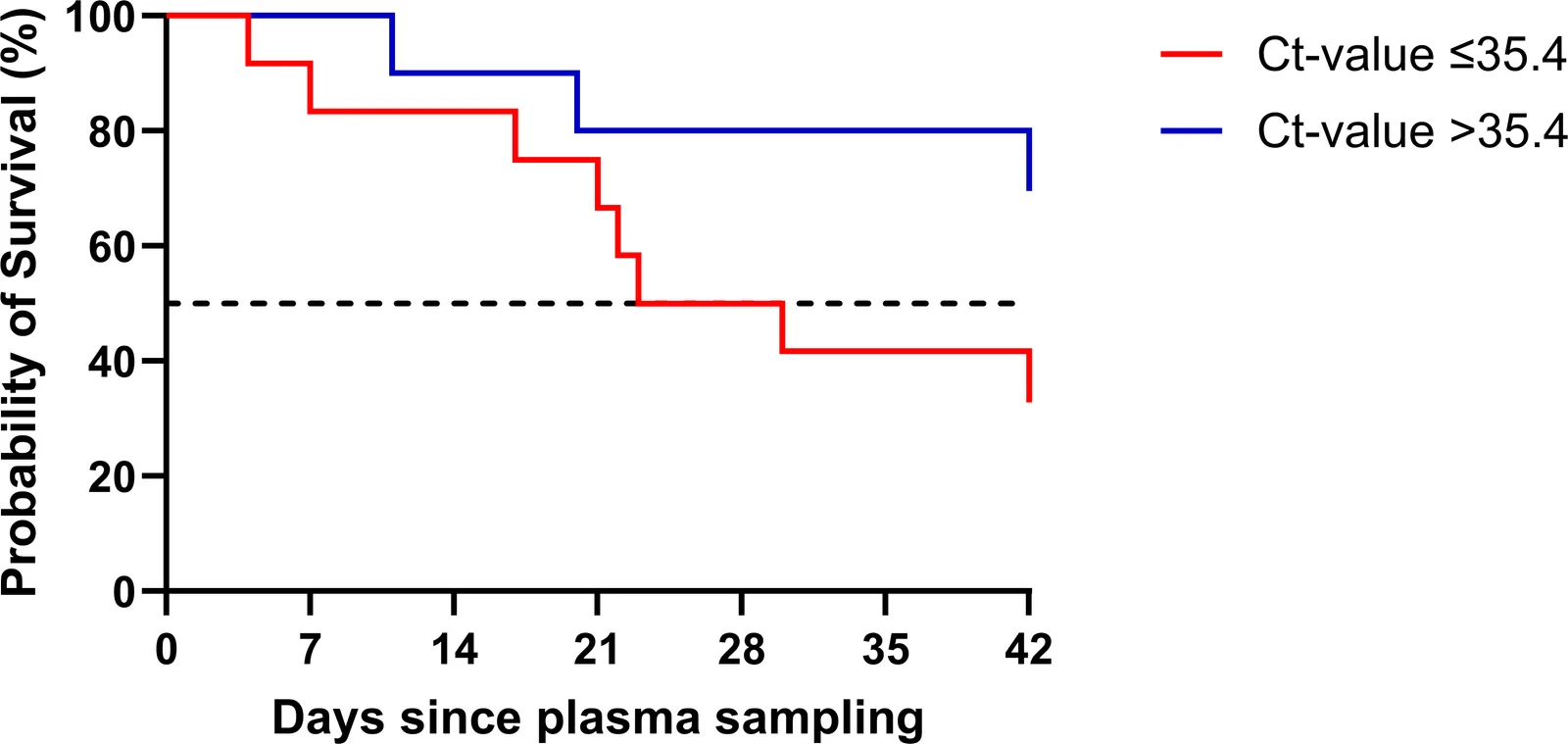
Six-week survival of patients with a positive in-house *A. fumigatus* PCR on large plasma volume with Ct-value ≤ 35.4 (*n* = 12) and patients with a positive in-house *A. fumigatus* PCR on large plasma volume with Ct-value > 35.4 (*n* = 10), Log-Rank *P* = 0.097.

Lastly, we wanted to evaluate the correlation of positive results of large-volume plasma PCR with 6-week mortality. As we used the Ct-values, we only included patients with a positive *A. fumigatus* PCR. For 6-week mortality, the area under the ROC curve of *A. fumigatus* PCR on large plasma volume was 0.649 (95% CI 0.414–0.883); see also Fig. S2 and Table S2. The Ct-value cut-off for optimal discrimination was 35.4, with a KS metric and Youden’s *J* statistic of 0.364, a sensitivity of 72.7%, and a specificity of 63.6%. When stratified to this Ct-value, the 6-week mortality was 66.7% (8/12) in patients with lower Ct-values, and 30.0% (3/10) in patients with higher Ct-values; the Kaplan-Meier curve is displayed in Fig. 2, *P* = 0.097. When interpreting these results, it is important to consider that this analysis is limited to patients with a positive plasma *A. fumigatus* PCR result, in this case in 22/110 (20%) samples tested.

### Diagnostic performance of PCR on plasma-derived exosomes

Finally, we performed PCR on plasma-derived exosomes in a convenience sample of 25 samples with sufficient plasma available from patients with suspected IA; all internal controls were successful. For *A. fumigatus*, 5/25 samples tested positive (20.0%), with a sensitivity of 29.4%, specificity 100%, PPV 100%, and NPV 40.0%. For *A. flavus*, 1/25 patients tested false positive (4.0%), while for *A. terreus* no positive test results were obtained.

## DISCUSSION

### Main findings

In this cohort of hematology patients with suspected IA, large plasma input volumes significantly improved the sensitivity of *A. fumigatus* PCR (52.6%) compared with small volumes (29.7%) and plasma-derived exosomes (29.4%), while maintaining high specificity (97.2%) and PPV (90.9%). Sensitivity increased to 67% in patients not receiving mold-active antifungal prophylaxis or therapy at plasma sampling. Lower Ct-values were associated with poorer 6-week survival (≤35.4 66.7% vs >35.4 30.0%, *P* = 0.097), suggesting that fungal DNA burden may aid risk stratification. However, this requires further confirmation.

### Clinical relevance

Diagnostic tests with a high NPV can help to exclude IA and reduce unnecessary empirical antifungal treatment, particularly in low-prevalence settings. In our cohort (IA prevalence of 34.2%), the NPV of large-volume plasma PCR was moderate (79.5%), indicating that it cannot reliably exclude IA as a standalone test. Its clinical value therefore lies primarily in improving diagnostic yield, particularly in patients with negative serum GM but persistent clinical suspicion of IA. Sensitivity declined after ≥72 h of antifungal therapy, emphasizing the importance of early sampling to minimize false negatives. Multimodal strategies combining plasma PCR, GM, and imaging are essential to improve diagnostic accuracy and support pre-emptive invasive fungal infection strategies (36–38), not only for IA but also for mucormycosis (39–41).

### Strengths and limitations

This study’s strengths include the novel and systematic comparison of three test modalities in a high-risk cohort with prospectively collected plasma samples. The β-globin internal control confirmed efficient DNA recovery without inhibition from larger plasma volumes.

An important limitation is that the estimated sensitivity of the *A. fumigatus* plasma PCR depends on the EORTC/MSGERC reference standard, which may not always accurately classify true infection. Several cases of probable IA were based on galactomannan alone, and species-level confirmation was lacking, leaving uncertainty about possible cross-reactive non-*Aspergillus* infections. Infections caused by cryptic *Aspergillus* species not targeted by the assay may also have reduced the estimated sensitivity. The sensitivity and specificity of the large-volume PCR for *A. flavus* could not be assessed because no IA cases caused by *A. flavus* were identified. Fungal culture and BAL PCR remained negative in all five patients with detectable *A. flavus* DNA, while *A. flavus* DNA was also detected in plasma from three healthy controls. These findings indicate that a positive *A. flavus* plasma PCR should be interpreted cautiously. Excluding patients with incomplete or inconclusive mycological testing improved internal validity by creating a fully evaluable non-IA comparator group. However, the heterogeneous “possible IA” category remains part of routine practice and should be considered when interpreting specificity estimates.

The limited sensitivity of exosome-derived DNA testing may reflect a low abundance of *Aspergillus*-derived nucleic acids within exosomes. Extracellular vesicle cargo has been characterized more extensively for RNA than DNA across fungal species (42–44), whereas data for *Aspergillus*, particularly from clinical samples, remain limited. The relatively low plasma input volume and the multi-step, precipitation-based enrichment may also have reduced DNA recovery, although exosome characterization and DNA yield were not assessed.

A substantial proportion of patients (39%) were not neutropenic at plasma sampling. Because serum galactomannan is less sensitive in non-neutropenic patients (45), plasma PCR sensitivity may likewise have been reduced. The low frequency of positive BAL fluid culture, as expected in patients with underlying hematological disease, may also have resulted in misclassification of IA, potentially underestimating both the sensitivity and specificity of plasma PCR.

Finally, Ct-value-based analyses were intrinsically restricted to positive tests, limiting the strength and generalizability of prognostic interpretations.

### Recommendations for clinical practice and future research

Large-volume plasma PCR *for A. fumigatus* provided modest but significant improvement in sensitivity without compromising specificity. Standardizing internal controls and pre-analytical workflows remains essential to minimize variability. Future studies should validate large-volume plasma PCR in lower-prevalence settings and assess its impact on antifungal stewardship. A major reduction in empirical antifungal therapy is unlikely because the NPV is insufficient to safely exclude IA. Instead, earlier detection of *Aspergillus* DNA in patients with negative conventional mycological tests but ongoing clinical suspicion will convince the clinician that triazole therapy is appropriate rather than to continue broad-spectrum (combination) antifungal therapy. Longitudinal studies of DNA kinetics may further refine prognostic models and guide treatment decisions.

### Conclusion

Large-volume plasma PCR for *A. fumigatus* significantly improves sensitivity compared to small plasma volumes and plasma-derived exosomes, while maintaining specificity and PPV. Sensitivity decreases after antifungal therapy, and Ct-values may provide prognostic insight.

## Data Availability

The data that support the findings of this study are available on request from the corresponding author. The data are not publicly available due to privacy and ethical restrictions.

## SUPPLEMENTAL MATERIAL

Supplemental Material jcm.00703-26-s0001.docx Fig. S1 and S2; Tables S1 and S2.

ASM does not own the copyrights to Supplemental Material that may be linked to, or accessed through, an article. The authors have granted ASM a non-exclusive, world-wide license to publish the Supplemental Material files. Please contact the corresponding author directly for reuse.
