# Supplementary material for: Performance of large-volume plasma PCR for the diagnosis of invasive aspergillosis: a multicenter retrospective cohort study
Source: J Clin Microbiol. 2026 Sep 25;64(10):e00703-26. doi: 10.1128/jcm.00703-26 (PMC13712825; doi:10.1128/jcm.00703-26)
Supplement: Supplementary file 1 [file jcm.00703-26-s0001.docx]

**Supplementary Figure 1**. Overview of the selection of samples tested.

**Supplementary Figure 2.** ROC-curve for the Ct-values of *A. fumigatus* PCR on large plasma volume (Mdn 4.0 ml, n=22) and 6-week mortality, with AUC 0.649 (95%CI 0.414-0.883). The Ct-value of 35.35, which optimally discriminates between 6-week mortality and survival, is indicated by an asterisk.

| **Sample number** | **Plasma input volume (ml)** | **Measured Ct-value decrease for β-globin** | **Predicted Ct-value decrease for β-globin** | **Difference between calculated and predicted Ct-value for β-globin** |
| --- | --- | --- | --- | --- |
| 1 | 4.0 | 7.2 | 3.7 | 3.5 |
| 2 | 4.0 | 6.7 | 3.7 | 3.0 |
| 3 | 4.0 | 6.6 | 3.7 | 2.9 |
| 4 | 4.0 | 5.7 | 3.7 | 2.0 |
| 5 | 3.0 | 5.1 | 3.3 | 1.8 |
| 6 | 4.0 | 5.3 | 3.7 | 1.6 |
| 7 | 3.0 | 4.7 | 3.3 | 1.4 |
| 8 | 2.3 | 4.2 | 2.9 | 1.3 |
| 9 | 4.0 | 4.9 | 3.7 | 1.2 |
| 10 | 2.0 | 3.9 | 2.7 | 1.2 |
| 11 | 2.0 | 3.9 | 2.7 | 1.2 |
| 12 | 3.0 | 4.4 | 3.3 | 1.1 |
| 13 | 3.0 | 4.4 | 3.3 | 1.1 |
| 14 | 3.0 | 4.3 | 3.3 | 1.0 |
| 15 | 4.0 | 4.6 | 3.7 | 0.9 |
| 16 | 4.0 | 4.4 | 3.7 | 0.7 |
| 17 | 4.0 | 4.4 | 3.7 | 0.7 |
| 18 | 4.0 | 4.4 | 3.7 | 0.7 |
| 19 | 4.0 | 4.3 | 3.7 | 0.6 |
| 20 | 4.0 | 4.3 | 3.7 | 0.6 |
| 21 | 2.3 | 3.5 | 2.9 | 0.6 |
| 22 | 2.0 | 3.2 | 2.7 | 0.5 |
| 23 | 4.0 | 4.0 | 3.7 | 0.3 |
| 24 | 2.0 | 3.0 | 2.7 | 0.3 |
| 25 | 4.0 | 3.8 | 3.7 | 0.1 |
| 26 | 4.0 | 3.8 | 3.7 | 0.1 |
| 27 | 3.0 | 3.3 | 3.3 | 0.0 |
| 28 | 2.0 | 2.7 | 2.7 | 0.0 |
| 29 | 3.0 | 3.1 | 3.3 | -0.2 |
| 30 | 3.0 | 2.9 | 3.3 | -0.4 |
| 31 | 2.2 | 1.0 | 2.9 | -1.9 |
| 32 | 4.0 | 0.9 | 3.7 | -2.8 |

**Supplementary Table 1.** Observed differences in β-globin Ct values between large and small plasma volumes, and corresponding predicted Ct shifts. Predicted Ct differences were calculated using the formula below, with the 1.5-fold factor derived from the difference in elution volumes (50 µL for the MagNA Pure 96 System vs 75 µL for the Maxwell® RSC Instrument). In only 2/32 samples (6.3%), the observed reaction efficiency and DNA recovery were substantially lower than expected.

$\frac{Ln (\frac{\frac{large volume used (ml)}{conventional volume used (ml)}}{1.5})}{Ln (2)}$

| **Ct-values** | **Sensitivity (%)** | **Specificity (%)** |
| --- | --- | --- |
| 29.7 | 0.0 | 100.0 |
| 31.4 | 9.1 | 100.0 |
| 32.3 | 18.2 | 100.0 |
| 32.6 | 18.2 | 90.9 |
| 32.8 | 27.3 | 90.9 |
| 33.5 | 27.3 | 81.8 |
| 34.1 | 36.4 | 81.8 |
| 34.2 | 36.4 | 72.7 |
| 34.4 | 45.5 | 72.7 |
| 34.5 | 45.5 | 63.6 |
| 34.8 | 54.5 | 63.6 |
| 35.2 | 63.6 | 63.6 |
| 35.4 | 72.7 | 63.6 |
| 35.7 | 72.7 | 54.5 |
| 35.9 | 72.7 | 45.5 |
| 36.0 | 72.7 | 27.3 |
| 36.1 | 81.8 | 27.3 |
| 36.3 | 90.9 | 27.3 |
| 36.7 | 100.0 | 18.2 |
| 37.8 | 100.0 | 9.1 |
| 39.8 | 100.0 | 0.0 |

**Supplementary Table 2.** The coordinates of the ROC curve of *A. fumigatus* PCR on large plasma volume and 6-week mortality from which sensitivity and specificity were derived for all the Ct-values of a positive PCR result.
